# *Botryococcus terribilis* Ethanol Extract Exerts Anti-inflammatory Effects on Murine RAW264 Cells

**DOI:** 10.3390/ijms24076666

**Published:** 2023-04-03

**Authors:** Shinya Takahashi, Farhana Ferdousi, Seri Yamamoto, Atsushi Hirano, Sachiko Nukaga, Hiroyuki Nozaki, Hiroko Isoda

**Affiliations:** 1 Faculty of Life and Environmental Sciences, University of Tsukuba, Tsukuba 305-8572, Japan; 2 Alliance for Research on the Mediterranean and North Africa (ARENA), University of Tsukuba, Tsukuba 305-8572, Japan; 3 Graduate School of Science and Technology, University of Tsukuba, Tsukuba 305-8572, Japan; 4 Tokyo Electric Power Company Holdings, Inc., Tokyo 100-8560, Japan

**Keywords:** anti-inflammation, *Botryococcus*, microalgae, RAW264 cells, AXL inhibitor, DNA microarray

## Abstract

The present study aimed to evaluate the effects of *Botryococcus terribilis* ethanol extract (BTEE) on lipopolysaccharide (LPS)-induced inflammation in RAW264 cells. BTEE significantly attenuated LPS-induced nitric oxide production and inflammatory cytokines release, including *Ccl2*, *Cox2*, and *Il6*. On the other hand, several anti-inflammatory mediators, such as *Pgc1β* and *Socs1*, were increased in BTEE-treated cells. Further, we performed an untargeted whole-genome microarray analysis to explore the anti-inflammatory molecular mechanism of BTEE. Enrichment analysis showed BTEE significantly downregulated ‘response to stimulus’, ‘locomotion’, and ‘immune system response’ and upregulated ‘cell cycle’ gene ontologies in both 6- and 17-h post-LPS stimulation conditions. Pathway analysis revealed BTEE could downregulate the expressions of chemokines of the CC and CXC subfamily, and cytokines of the TNF family, TGFβ family, IL1-like, and class I helical. PPI analysis showed AXL receptor tyrosine kinase (*Axl*), a receptor tyrosine kinase from the TAM family, and its upstream transcription factors were downregulated in both conditions. Node neighborhood analysis showed several *Axl* coexpressed genes were also downregulated. Further, kinase enrichment and chemical perturbation analyses supported *Axl* inhibition in BTEE-treated conditions. Altogether, these findings suggest anti-inflammatory effects of BTEE that are mediated via the suppression of pro-inflammatory cytokines and predict its potential as an *Axl* inhibitor.

## 1. Introduction

Inflammation is a biological defense response that protects the host from various stimuli, such as invading infectious microorganisms—bacteria and viruses, physical stimuli—burns and frostbite, and ultraviolet light [1].

In mammals, there are two types of inflammation: acute and chronic [2,3]. Acute inflammation is short-lived and disappears when the role of the inflammatory response is over. However, if that acute inflammation persists or the substances that promote inflammation are not removed, it can become chronic. Chronic inflammation is not a specific disease but a mechanistic process that can lead to long-term tissue destruction and functional impairment. It is a crucial factor causing almost all potentially disabling or life-threatening illnesses, including cardiovascular diseases, diabetes, cancer, arthritis, neurological diseases, and bowel diseases. Therefore, it is essential to prevent chronic inflammation to reduce the risk of chronic diseases and improve quality of life. 

The most conventional drugs used for managing chronic inflammation include over-the-counter nonsteroidal anti-inflammatory drugs (NSAIDs) and prescribed drugs—corticosteroids, statins, etc. However, gastrointestinal, cardiovascular, hepatic, renal, brain, and pulmonary side effects of NSAIDs and steroids are well-known [4,5]. Therefore, in recent years, much attention has been devoted to exploring anti-inflammatory natural products and dietary supplements with fewer side effects that may remove inflammation triggers and alleviate inflammation-induced chronic diseases [6,7].

Recently, algae have been reported to produce a variety of compounds, such as polysaccharides, lipids, proteins, and pigments [8]. Algae are photosynthetic organisms that grow in various habitats, including lakes, rivers, and sewage systems. Large algae (seaweeds) and microalgae (single cells) are the two main categories of algae. It is also worth mentioning that algae can be mass-produced. Moreover, they can withstand a wide range of temperatures, salinities, and pH levels [8]. Because of these characteristics, algae have the potential to be a valuable natural resource for novel bioactive compounds.

Several bioactivities have been found in many species of algae. For example, the *Aurantiochytrium mangrovei* 18W-13a and *Aurantiochytrium limacinum* 4W-1b contain several bioactive substances, such as squalene, and have anti-inflammatory properties [9,10]. In addition, carotenoids from *Haematococcus pluvialis* and *Dunaliella salina*, and hydrocarbons from *Chlorella vulgaris* and *Phaeodactylum tricornutum*, have been reported to have anti-inflammatory properties [11,12,13,14].

*Botryococcus*, a type of freshwater green microalgae, can accumulate large amounts of hydrocarbons. While its potential as a petroleum substitute is garnering attention, it has also been discovered to be beneficial as a useful bioresource. Recently, microalgal extract from the green algae, *Botryococcus braunii*, has been shown to promote neurogenesis in mice by stimulating energy production, exerting anti-stress and anti-depressant effects, and suppressing neuroinflammation. [15]. *Botryococcus terribilis* (BT), a recently identified *Botryococcus* strain, is one of the most common species in Cuba and southern Spain, with a tropical distribution [16]. HPLC analysis of the BT extract identified two major compounds: methylated-meijicoccene, a new molecule, and C32 botryococcene, a triterpene [17]. In BT extract, methylated-meijicoccene is present abundantly at 42.7%. Our earlier studies have shown that BT and its components may promote hair growth [17] and exhibit antidepressant-like properties [18]. However, its comprehensive bioactive potential remains to be explored. 

In this present study, we investigated the anti-inflammatory effects of ethanol extract of BT (BTEE) in lipopolysaccharide (LPS)-stimulated RAW264 cells, a macrophage-like cell, originating from Abelson leukemia virus-transformed cell line derived from BALB/c mice [19]. RAW264 cells exhibit a reasonably stable, mature, and adherent macrophage phenotype, a high proliferation rate, and maintain functional characteristics such as nitric oxide (NO) production in response to LPS stimulation for up to 30 successive passages [20]. Therefore, RAW 264 cells have been widely used for preliminary screening of natural products with anti-inflammatory activities and to predict their prospective effect in vivo or on primary cells. We further performed an untargeted whole-genome transcriptomic analysis on BTEE-treated and LPS-stimulated RAW264 cells for the prediction of the interaction of BTEE on possible molecular targets and biological pathways.

## 2. Results

### 2.1. Cytotoxic Effect of BTEE on RAW264 Cells

**Figure 1:**
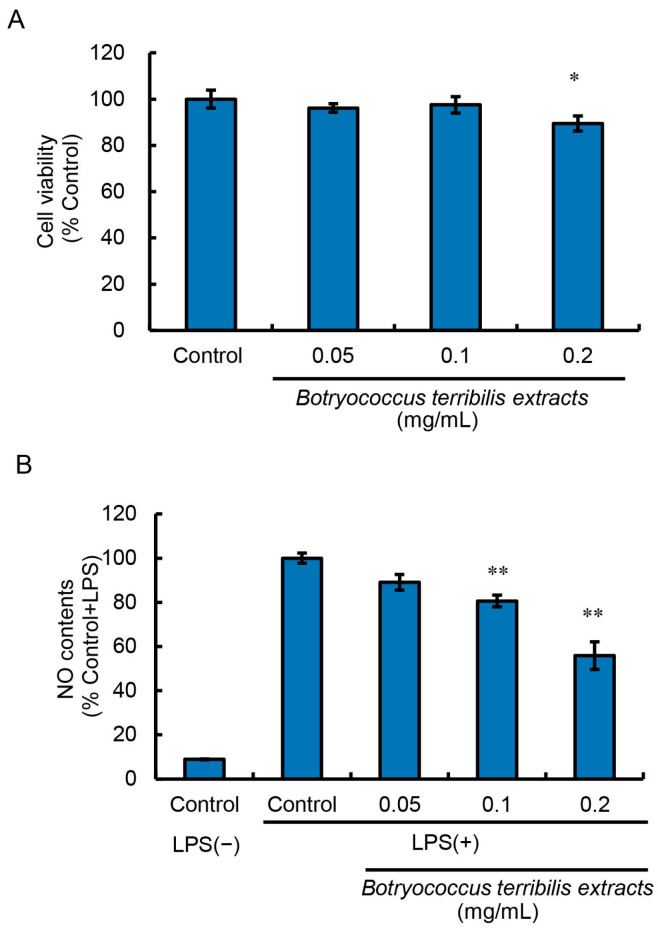
Effect of BTEE on cell viability and LPS-induced NO production in RAW264 cells. (**A**) RAW264 cells were treated with or without BTEE at the concentrations of 0.05, 0.1, and 0.2 mg/mL for 24 h. After treatment, cell viability was measured by MTT assay. Values are expressed as mean ± SD of triplicate experiments and as relative to the percentage of control. (**B**) Cells were treated with or without BTEE at concentrations of 0.05, 0.1, and 0.2 mg/mL for 24 h. After treatment, cells were stimulated with LPS (1 ng/mL) for 16 h. The amount of NO production was measured by the Griess reaction. Values are expressed as mean ± SD of triplicate experiments and as relative to the percentage of control LPS (+). Significance is indicated as * *p* < 0.05 or ** *p* < 0.01.

RAW264 cells were treated with BTEE diluted at 1/2000 (0.05 mg/mL), 1/1000 (0.1 mg/mL), and 1/500 (0.2 mg/mL). The cytotoxicity of BTEE was evaluated using the MTT assay. BTEE showed no significant cytotoxicity up to the concentration of 0.1 mg/mL (Figure 1A).

### 2.2. Effect of BTEE on LPS-Induced Nitric Oxide Production

Nitric oxide (NO) is a signaling molecule that plays a critical role in the immune system [21]. The overproduction of NO has been implicated in a variety of pathophysiological conditions [22]. Therefore, suppression of NO production has become an important target in treating inflammation. Consequently, we examined the effect of BTEE on LPS-induced NO production.

Upon LPS stimulation, NO production was not significantly increased until 6 h; however, NO content significantly increased after 16 h of LPS stimulation. Therefore, we examined the effects of BTEE on NO production after 16 h of LPS stimulation.

We found that BTEE pretreatments for 24 h at the dilutions of 1/2000 (0.05 mg/mL), 1/1000 (0.1 mg/mL), and 1/500 (0.2 mg/mL) could significantly reduce NO production 16 h post-LPS stimulation in a dose-dependent manner (Figure 1B).

### 2.3. Effects of BTEE on Gene Expression of Inflammatory Mediators

As described above, BTEE was found to have an anti-inflammatory effect. Therefore, we next evaluated whether pretreatment with BTEE could alter the expression of representative inflammation-related mediators, C-C motif chemokine ligand 2 (*Ccl2*), interleukin 1 beta (*Il1b*), inducible nitric oxide synthase (*iNos*), cyclooxygenase 2 (*Cox2*)*,* tumor necrosis factor (*Tnf*), and interleukin 6 (*Il6*), in RAW264 cells. 

**Figure 2:**
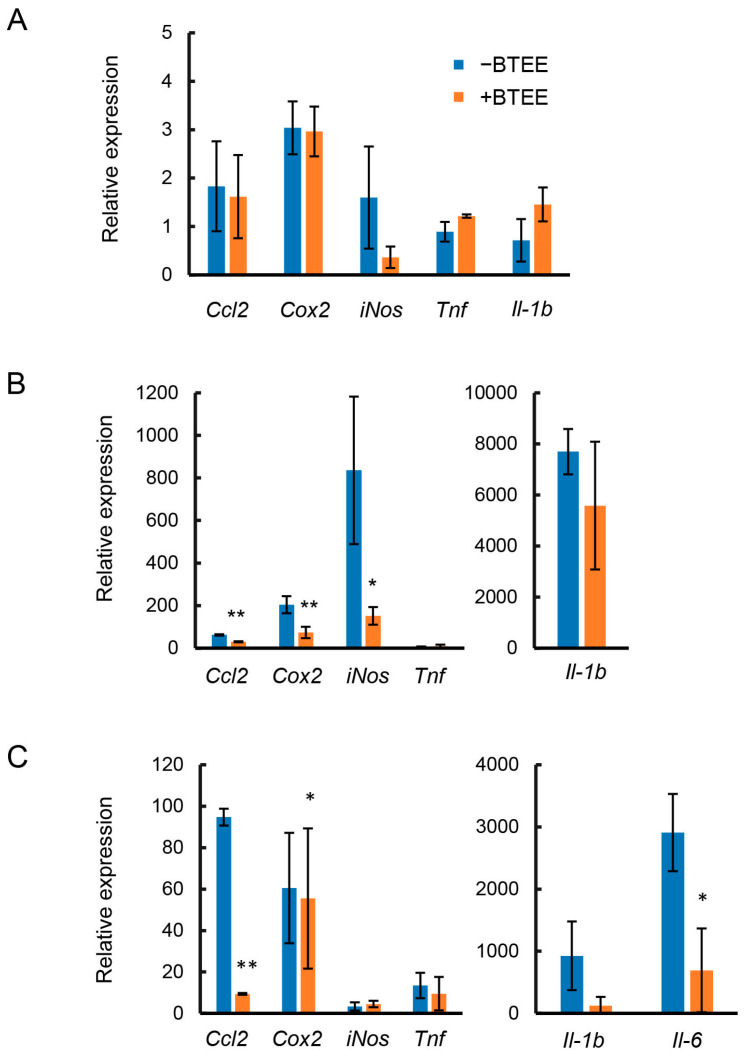
Effects of BTEE on the mRNA expression of inflammatory mediators. Cells were treated with or without BTEE at concentrations of 0.2 mg/mL for 24 h. After treatment, cells were activated with LPS (1 ng/mL) for (**A**) 0 h, (**B**) 6 h, and (**C**) 17 h. The mRNA expressions of pro-inflammatory mediators were determined by RT-PCR. Values are expressed as the mean ± SD of triplicate experiments. Significance is indicated as * *p* < 0.05 or ** *p* < 0.01.

Before LPS stimulation, no significant difference was observed in *Ccl2*, *Cox2*, *iNos*, *Tnf*, and *Il1b* expression between control (BTEE-) and BTEE-treated (BTEE+) conditions (Figure 2A). At 6 h of LPS stimulation, there was no expression of *Il6*. In addition, there was no change in *Tnf* expression. Significant decreases were observed in *Ccl2* and *iNos* expressions and a decreasing trend was observed in *Il1β* expression (Figure 2B). At 17 h of LPS stimulation, *Ccl2*, *Cox2*, *Tnf*, and *Il6* were significantly decreased in the BTEE-treated cells compared to control cells (Figure 2C). *Il1b* showed a decreasing trend (Figure 2C). However, there was no change in *iNos* expression. Previous studies demonstrated that the mRNA expression of iNos reaches its peak from 6 to 8 h after LPS stimulation and then gradually decreases with time [23,24], which may explain the very low expression of *iNos* at 17 h post-LPS stimulation in our study (Figure 2C). Additionally, in RAW264 cells, iNOS mRNA and protein expressions are found to diminish before NO content reaches its maximum. A high level of NO content is found long after iNOS protein and mRNA expressions begin to decline [24], which is consistent with our observation of high NO content 16 h after LPS stimulation (Figure 1B).

### 2.4. Effects of BTEE on mRNA Expressions of Anti-Inflammatory Mediators

As described above, treatment with the BTEE on RAW264 cells suppressed the gene expressions of inflammatory mediators. Therefore, we next evaluated whether pretreatment with BTEE could alter the gene expressions of representative anti-inflammatory mediators, peroxisome proliferator-activated receptor-gamma coactivator 1 beta (*Pgc1b*), activating transcription factor 3 (*Atf3*), and suppressing cytokine signaling 1 (*Socs1*), in RAW264 cells.

**Figure 3:**
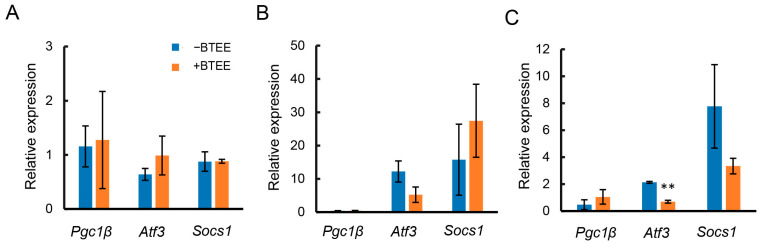
Effects of BTEE on the mRNA expressions of anti-inflammatory mediators. Cells were treated with or without BTEE at concentrations of 0.2 mg/mL for 24 h. After treatment, cells were stimulated with LPS (1 ng/mL) for (**A**) 0 h, (**B**) 6 h, and (**C**) 17 h. The mRNA expressions of pro-inflammatory mediators were determined by RT-PCR. Values are expressed as the mean ± SD of triplicate experiments. ** *p* < 0.01 significance compared to control samples.

Before LPS stimulation, there was no significant difference in the expressions of *Pgc1b*, *Atf3*, and *Socs1* (Figure 2A). At 6 h of LPS stimulation, *Atf3* tended to decrease, *Pgc1b* remained unchanged, and *Socs1* tended to increase in BTEE-treated cells compared to control cells (Figure 3B).

At 17 h of LPS stimulation, *Atf3* was significantly decreased, *Socs1* showed a decreasing trend, and *Pgc1b* showed an increasing trend in the BTEE-treated condition compared to the nontreated condition (Figure 3C).

### 2.5. Characteristics of Gene Expression Profiling in BTEE-Treated RAW264 Cells

We performed whole-genome DNA microarray analysis to investigate the changes in gene expression in LPS-stimulated RAW264 cells treated with BTEE for 6 or 17 h. 

**Figure 4:**
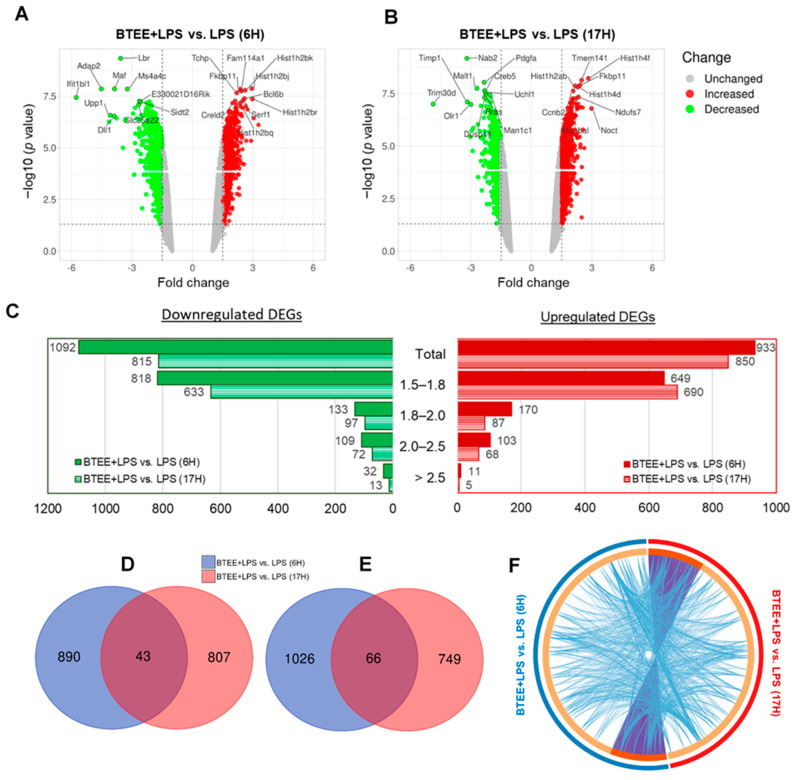
Characterization of gene expression profile in BTEE-treated RAW264 cells. Volcano plots displaying differentially expressed genes (DEGs) between BTEE-treated and nontreated RAW264 cells (**A**) 6 h after LPS induction and (**B**) 17 h after LPS induction. The vertical axis (y-axis) corresponds to −log10 *p* value and the horizontal axis (x-axis) displays linear fold change. The red dots represent the upregulated genes; the green dots represent the downregulated genes. The top 20 DEGs with the biggest fold changes are shown. (**C**) Bar graphs showing the distribution of fold changes of the DEGs in BTEE-treated RAW264 cells. The red bars (red fill bars refer to 6 h and pattern bars refer to 17-h condition) represent the numbers of upregulated DEGs; the green bars represent the numbers of downregulated genes (green fill and pattern bars refer to 6 h and 17-h conditions, respectively). Venn diagrams illustrate gene expression similarity between two experimental conditions in RAW264 cells. Blue circles represent DEGs between BTEE + LPS vs. LPS 6 h and red circles represent BTEE + LPS vs. LPS 17-h conditions; (**D**) Upregulated DEGs and (**E**) Downregulated DEGs. (**F**) The Circos plot shows how genes overlap between 6 h and 17-h conditions. On the outside, the blue arc represents the DEG list from BTEE + LPS vs. LPS 6 h and the red arc represents the DEG list from BTEE + LPS vs. LPS 17-h condition. On the inside, the dark orange color represents the DEGs shared by both 6-h and 17-h conditions and the light orange color represents the DEGs unique to each condition. Purple lines link the common DEGs shared by both conditions. Blue lines connect the DEGs that fall under the same significantly enriched gene ontology term.

A total of 21,978 genes were identified by the Clariom S assay (mouse). After 6 h of LPS stimulation, 2350 genes were differentially expressed in the BTEE-treated group compared to the nontreated group. After deleting duplicates and ‘no symbol’ genes, 2026 differentially expressed genes (DEGs) were identified; among them, 1092 DEGs were downregulated and 933 DEGs were upregulated (Figure 4A). At 17 h of LPS stimulation, 1665 DEGs were identified in the BTEE-treated group compared to the nontreated group; among them, 815 DEGs were downregulated and 850 were upregulated (Figure 4B). Figure 4C shows the distribution of fold changes in 6 h and 17-h conditions. A total of 43 genes were commonly upregulated (Figure 4D) and 66 genes were commonly downregulated (Figure 4E) in both 6 h and 17-h conditions. A complete list of common DEGs is provided in Supplementary File S1. Although there were not many overlapped DEGs between 6 h and 17 h treatment conditions, there was significantly more functional overlap (Figure 4F).

### 2.6. Gene Ontology and Pathway Enrichment Analyses of the BTEE-Induced RAW264 Cells

**Figure 5:**
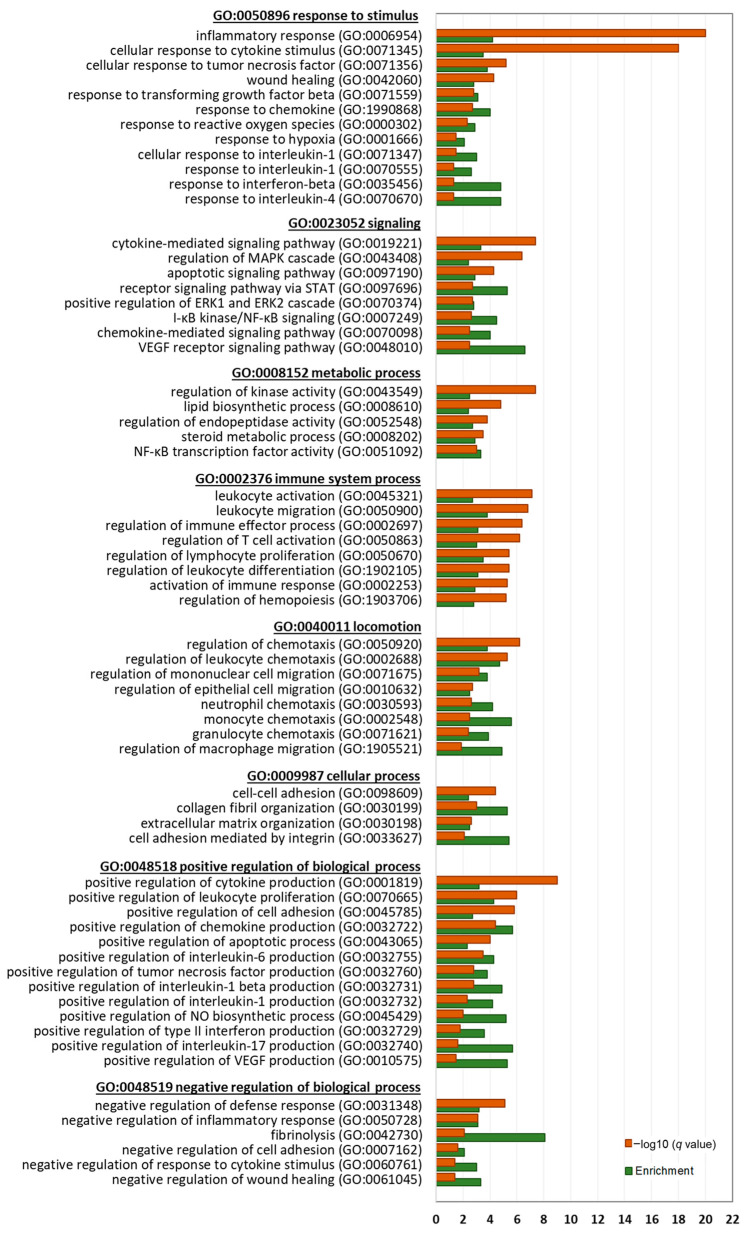
Biological process gene ontology (GOBP) analysis of downregulated DEGs between BTEE-treated and nontreated RAW264 cells after 6 h LPS stimulation. Top enriched GOBP terms are presented under their parent terms. The orange bars represent the −log10 *q* value (Benjamini–Hochberg *p* value correction algorithm for multiple comparisons) and the green bars represent the enrichment score.

**Figure 6:**
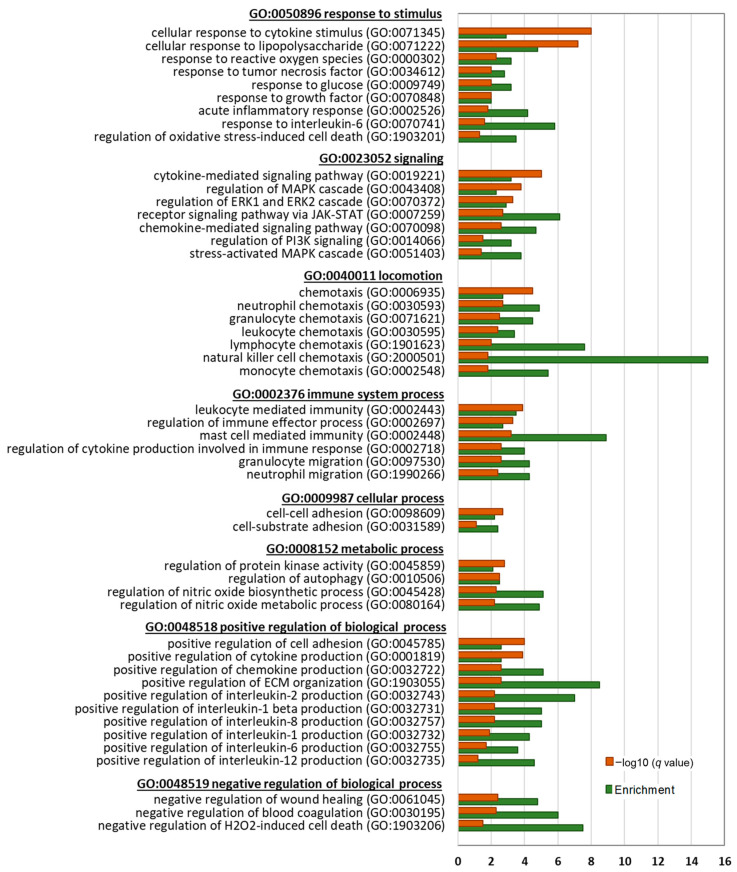
Biological process gene ontology (GOBP) analysis of downregulated DEGs between BTEE-treated and nontreated RAW264 cells after 17 h LPS stimulation. Top enriched GOBP terms are presented under their parent terms. The orange bars represent the −log10 *q* value (Benjamini–Hochberg adjusted *p* value for multiple comparisons), and the green bars represent the enrichment score.

Next, we performed a detailed gene ontology (GO) analysis of significantly enriched biological processes (BP) by the DEGs to identify key biological themes regulated by BTEE (Figure 5 and Figure 6). 

Downregulated DEGs at both 6-h and 17-h conditions significantly enriched parent GOBP terms of response to stimulus (GO:0050896), signaling (GO:0023052), locomotion (GO:0040011), immune system process (GO:0002376), cellular process (GO:0009987), metabolic process (GO:0008152), positive regulation of biological process (GO:0048518), and negative regulation of biological process (GO:0048519). Genes related to cellular response to cytokine stimulus (GO:0071345), lipopolysaccharide (GO:0071222), reactive oxygen species (GO:0000302), and interleukin 6 (GO:0070741) were downregulated. Further, genes that are positively associated with cytokine production (GO:0001819), chemokine production (GO:0032722), interleukin 2 production (GO:0032743), interleukin 1 beta production (GO:0032731), interleukin 8 production (GO:0032757), interleukin 1 production (GO:0032732), and interleukin 12 production (GO:0032735) were significantly downregulated. Additionally, NO metabolic process (GO:0080164) and NO biosynthetic process (GO:0045428)-related genes were downregulated.

Another top enriched parent BP by the downregulated DEGs was ‘locomotion’, which included chemotaxis of neutrophil (GO:0030593), granulocyte (GO:0071621), leukocyte GO:0030595), lymphocyte (GO:1901623), natural killer cell (GO:2000501), and monocyte (GO:0002548). Cell–cell adhesion (GO:0098609), negative regulation of wound healing (GO:0061045), and ECM organization (GO:1903055)-associated genes were also downregulated. 

Enriched signaling pathways by the downregulated DEGs included stress-activated MAPK cascade (GO:0051403), PI3K signaling (GO:0014066), receptor signaling pathway via JAK-STAT (GO:0007259), and ERK1 and ERK2 cascade (GO:0070372). 

**Figure 7:**
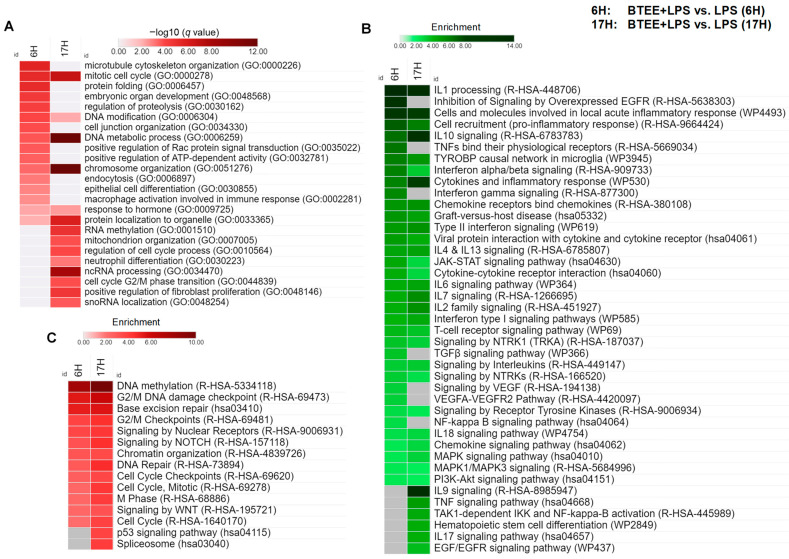
Biological process gene ontology (GOBP) and pathway enrichment analyses. (**A**) Heatmap shows significant GOBP terms in both 6-h and 17-h conditions enriched by the upregulated DEGs between BTEE-treated and nontreated RAW264 cells. Color code represents the −log10 *q* value (Benjamini–Hochberg adjusted *p* value for multiple comparisons). Significantly enriched pathways by the (**B**) downregulated and (**C**) upregulated DEGs. The color code represents the enrichment score.

On the other hand, the upregulated DEGs were associated with the mitotic cell cycle (GO:0000278), cell cycle process (GO:0010564), cell cycle G2/M phase transition (GO:0044839), as well as protein folding (GO:0006457), DNA modification (GO:0006304), DNA metabolic process (GO:0006259), chromosome organization (GO:0051276), RNA methylation (GO:0001510), and mitochondrion organization (GO:0007005) (Figure 7A).

Next, we performed pathway enrichment analysis to achieve mechanistic insight into gene lists overrepresented by BTEE (Figure 7B,C). We used multiple pathway databases, including Reactome, Kyoto Encyclopedia of Genes and Genomes (KEGG), and WikiPathways, to obtain both broad and detailed pathway terms. IL1 processing was the top enriched pathway by the downregulated DEGs in both 6 h and 17-h conditions. Several IL pathways, such as ILs 2, 4, 6, 7, 10, 13, and 18, were enriched in both conditions, whereas ILs 9 and 17 were enriched in only 17-h condition. TNFs, transforming growth factor beta (TGFβ), interferons (IFNs), chemokine, and T cell receptor signaling pathways were also enriched by the downregulated DEGs. Downregulated DEGs in BTEE-treated conditions related to ‘cytokine–cytokine receptor interaction’ KEGG pathway are shown in Supplementary Figure S1. Other important pathways include epidermal growth factor receptor (EGFR), vascular endothelial growth factor A (VEGF), neurotrophic tyrosine receptor kinase 1 (NTRK1), and nuclear factor-κB (NF-κB) pathways (Figure 7B).

Enriched pathways by the upregulated DEGs included DNA methylation, DNA damage checkpoints, chromatin organization, base excision repair, the cell cycle, as well as signaling by NOTCH, WNT, nuclear receptors, and the p53 signaling pathway (Figure 7C).

### 2.7. Common and Unique DEGs in BTEE-Treated 6-h and 17-h Conditions in RAW264 Cells

**Figure 8:**
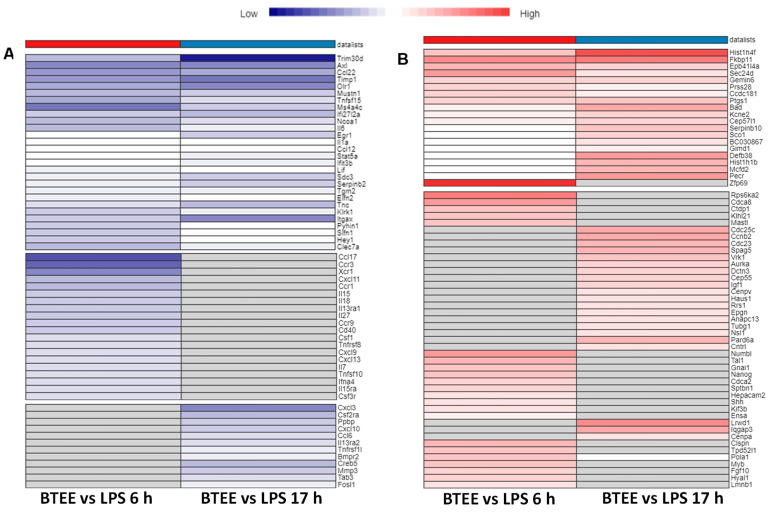
Heatmaps of DEGs of top enriched functions. (**A**) Expression profile of downregulated DEGs associated with several inflammatory pathways; (**B**) Expression profile of upregulated DEGs associated with cell cycle and cell division.

Key overlapped DEGs with enriched functions and their expressions are shown in heatmaps in Figure 8. Although most of the enriched GOBPs and pathways were similar in both 6-h and 17-h experimental conditions (as shown in Figure 5, Figure 6 and Figure 7), they did not always share the same DEGs (as shown in Figure 8).

Several inflammatory cytokines were downregulated in both 6-h and 17-h conditions; however, there were several unique inflammatory mediators to each condition (Figure 8A). Commonly upregulated DEGs were associated with the S phase of the cell cycle, whereas uniquely upregulated DEGs in both conditions were associated with the M phase of the cell cycle and mitotic cell division (Figure 8B).

### 2.8. Protein–Protein Interaction Network Analysis of the Commonly Regulated DEGs in 6-h and 17-h conditions in RAW264 Cells

We performed a protein–protein interaction (PPI) network analysis to uncover the interacting genes and to predict the molecular targets of BTEE’s biological functions.

**Figure 9:**
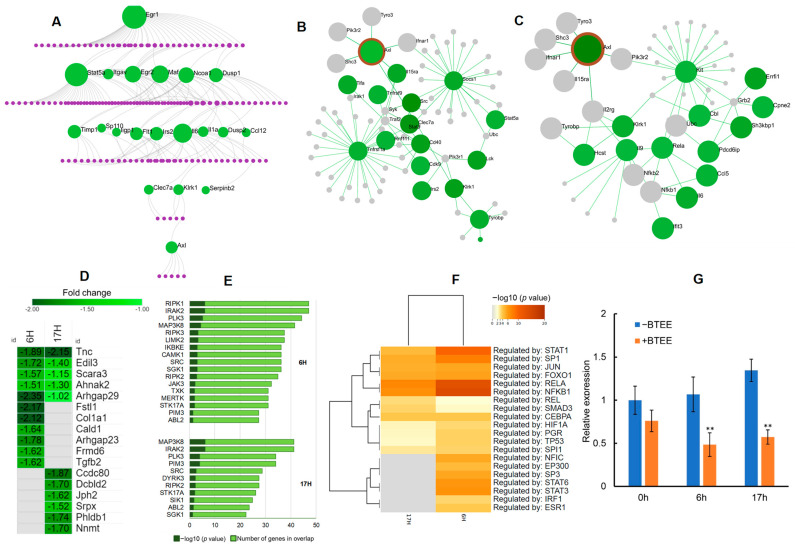
PPI interaction network and enrichment analysis. (**A**) Generic first-order PPI network by the commonly downregulated DEGs (*n* = 66) in both 6-h and 17-h conditions shown in the ‘Sugiyama’ layout. Each green node represents the seed proteins and edges indicating known interactions (InnateDB Interactome database) between two connecting proteins. Purple nodes represent nonsignificant genes. Node neighborhood analyses showing expression profile of highly interacting genes with *Axl* in (**B**) BTEE + LPS vs. LPS 6-h condition and (**C**) BTEE + LPS vs. LPS 17-h condition. (**D**) Heatmap showing expression profile of downregulated DEGs that coexpress with *Axl*. Coexpressed genes, ranked among the top 100, were identified with ARCHS4 gene–gene coexpression database. (**E**) Bar graph showing kinase enrichment analysis of the downregulated DEGs (ARCHS4 kinases coexpression database. (**F**) Heatmap showing TF enrichment analysis (TRRUST database). (**G**) Quantified PCR analysis data of *Axl*. ** *p* < 0.01 significance compared to control samples.

First, we built a first-order undirected PPI network using the InnateDB Interactome database [25] consisting of common downregulated DEGs (*n* = 66). PPI analysis identified 20 seeds and 179 interacting nodes with 199 edges (Figure 9A). Early growth response 1 (*Egr1*) was the top hub node with the highest degree (degree = 39, betweenness = 6279.43), followed by the signal transducer and activator of transcription 5A (*Stat5a*; degree = 34, betweenness = 8203.75), *Il6* (degree = 20, betweenness = 2958.51), MAF BZIP transcription factor (*Maf*; degree = 16, betweenness = 2594.85), *Egr2* (degree = 14, betweenness = 1688.33), and nuclear receptor coactivator 1 (*Ncoa1*; degree = 12, betweenness = 2012.67). Enriched networks included Th17 cell differentiation (FDR = 3.41 × 10^−27^), Jak-STAT signaling pathway (FDR = 6.24 × 10^−21^), IL17 signaling pathway (FDR = 9.76 × 10^−16^), TNF signaling pathway (FDR = 4.54 × 10^−14^), PI3K-Akt signaling pathway (FDR = 8.54 × 10^−13^), cytokine–cytokine receptor interaction (FDR = 4.50 × 10^−10^), MAPK signaling pathway (FDR = 1.22 × 10^−8^), T cell receptor signaling pathway (FDR = 1.87 × 10^−8^), and Toll-like receptor signaling pathway (FDR = 1.20 × 10^−7^). All hub nodes and network enrichment results are provided in Supplementary File S2.

Undirected PPI analysis of the common downregulated DEGs leads to identifying an interesting downstream seed gene, AXL receptor tyrosine kinase (*Axl*), a tyrosine kinase from the TAM family (Figure 9A). Therefore, we further performed neighborhood analyses of the seed gene *Axl* to examine the expression profile of genes highly connected to *Axl*. We found several genes interacting with *Axl* were downregulated in 6-h and 17-h conditions (Figure 9B,C).

Further, we expanded *Axl* with ‘All RNA-seq and ChIP-seq sample and signature search’ (ARCHS4) gene–gene coexpression matrix [26] by identifying the top 100 genes that mostly coexpress with *Axl*. Among the top 100 coexpressed genes, several were significantly downregulated in both 6-h and 17-h conditions (Figure 9D).

AXL has been reported to exhibit crosstalk with numerous receptor tyrosine kinases (RTKs). Additionally, most AXL inhibitors are known to inhibit other protein kinases, preferentially RTKs. Therefore, we performed a kinase enrichment analysis of the downregulated DEGs to explore the overrepresented kinases (ARCHS4 kinases coexpression database). Top enriched kinases by the downregulated DEGs included mixed lineage kinases (RIPK1, RIPK2, and RIPK3), interleukin-1 receptor-associated kinase 2 (IRAK2), receptor tyrosine kinases (TXK, MERTK), nonreceptor tyrosine kinases (JAK3, ABL2, SRC), mitogen-activated protein kinase (MAP3K8), IκB kinase family (IKBKE), and polo-like kinase (PLK) (Figure 9E). Details of kinase coexpression enrichment analysis can be found in Supplementary File S3. 

To explore other direct regulators of *Axl*, we performed a TF enrichment analysis using the TRRUST (transcriptional regulatory relationships unraveled by sentence-based text mining) database (Figure 9F) [27]. Top enriched TFs by the downregulated DEGs included TFs that directly act on the *Axl* promoter (SP1, SP3, and HIF1A), that regulate *Axl* mRNA expression through other signaling pathways (JUN), and that regulate important downstream pathways of *Axl* (STAT and NF-κB) [28]. 

### 2.9. Chemical Perturbations from LINCS L1000 Library by the Downregulated DEGs

**Figure 10:**
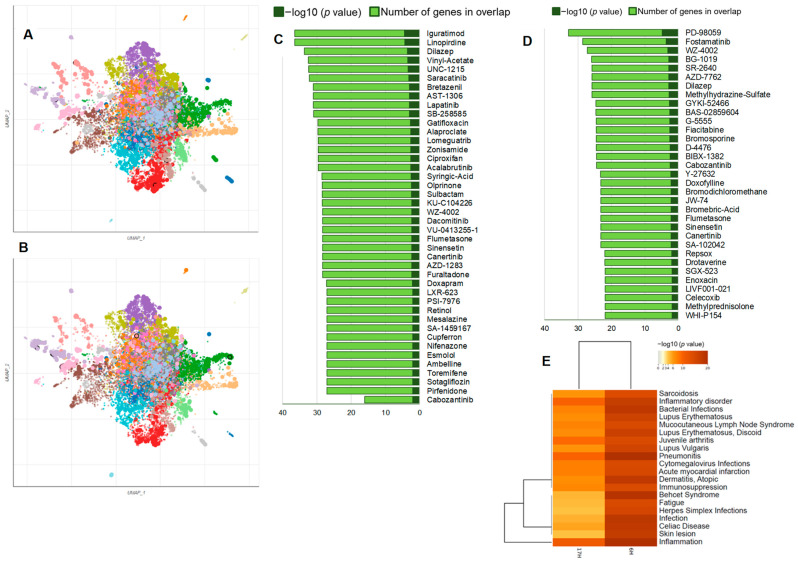
Chemical Perturbations and disease–gene association enrichment analysis *by downregulated DEGs in BTEE-treated RAW264 cells* from LINCS L1000 library and DisGeNET database. Scatterplots of all terms in the LINCS L1000 chemical perturbation consensus signature gene set library; (**A**) 6-h condition, (**B**) 17-h condition. Each point represents a term in the library. Term frequency–inverse document frequency (TF–IDF) values were computed for the gene set corresponding to each term, and the UMAP dimensionality reduction approach was applied to the resulting values. The terms are plotted based on the first two UMAP dimensions. Generally, terms with more similar gene sets are positioned closer together. Terms are colored by automatically identified clusters computed with the Leiden algorithm applied to the TF–IDF values. The darker and larger the point, the more significantly enriched the term. From the scatter plots, drugs or chemicals that downregulate *Axl* expression are identified and plotted in the bar graphs; (**C**) 6-h condition, (**D**) 17-h condition. (**E**) Heatmap shows enrichment analysis of disease–gene association in DisGeNET.

We have performed chemical/drug perturbations analysis using the LINCS L1000 library (Figure 10A–D) [29]. This analysis was limited to downregulated DEGs to examine the enrichment of *Axl* inhibitors. We identified several enriched chemicals/drugs in BTEE-treated conditions that not only downregulate *Axl* expression but also impact other genes through a complex network of both upstream and downstream regulatory interactions. For example, iguratimod, linopirdine, dilazep, vinyl-acetate, saracatinib, bretazenil, lapatinib, lomeguatrib, alaproclate, gatifloxacin, acalabrutinib, dacomitinib, and cabozantinib were enriched in 6-h condition, whereas fostamatinib, fiacitabine, dilazep, methylhydrazine-sulfate, bromosporine, cabozantinib, doxofylline, sinensetin, canertinib, celecoxib, and enoxacin were enriched in 17-h condition. Details of chemical perturbation enrichment analysis can be found in Supplementary File S4. These findings suggest that BTEE may have the potential to mimic the functions of the identified enriched drug or chemicals. 

Finally, we performed a disease–gene association enrichment analysis using the downregulated DEGs, which returned a list of diseases where BTEE is predicted to exert beneficial effects. Enriched disease terms included several inflammatory and autoimmune disorders as well as skin diseases (Figure 10E). The DisGeNET database was used for the disease–gene association enrichment analysis [30].

## 3. Discussion

BTEE exhibited anti-inflammatory effects that are mediated via the suppression of LPS-induced NO production and pro-inflammatory cytokines release. Further, an integrated whole-genome transcriptomic analysis predicted the interaction of BTEE on possible biological pathways and molecular targets.

Gene expression analysis showed that the expressions of six proinflammation-related genes, *Ccl2*, *Cox2*, *Tnf*, *iNos, Il1b,* and *Il6,* differed depending on the duration of LPS stimulation; however, BTEE treatment could overall decrease their expression. On the other hand, BTEE treatment upregulated to some extent the expressions of three anti-inflammation-related genes, *Atf3*, *Socs1,* and *Pgc1β,* at 6 h and 17 h post-LPS stimulation, respectively (Figure 1, Figure 2 and Figure 3).

Integrative evaluations of transcriptomics obtained from compound-induced gene expression data derived from biological experiments can capture the compound’s possible biological responses and molecular targets. Therefore, in the present study, we comprehensively analyzed how BTEE pretreatment affected the changes in gene expression in LPS-stimulated RAW264 cells. Enrichment analysis suggested that BTEE significantly downregulated gene expressions related to the cellular response to cytokine stimulus, LPS, and reactive oxygen species. More specifically, genes positively associated with cytokine production, such as *IL*s *1*, *2*, *8*, and *12*, and chemokine production, were downregulated in BTEE-treated cells. Additionally, signaling pathways of several tyrosine kinases—both receptor and nonreceptor—were downregulated, such as EGFR, VEGF, NTRK1, and NF-κB pathways (Figure 5, Figure 6 and Figure 7B). 

On the other hand, cell cycle, DNA methylation, DNA damage checkpoint, chromatin organization, as well as WNT, Notch, and p53 signaling pathways-related genes were significantly upregulated in BTEE-treated cells (Figure 7A,C).

Further, PPI and node neighborhood analyses predicted that BTEE interacts with *Axl* to exhibit its biological responses (Figure 9). First, untargeted PPI analysis of the commonly downregulated genes in both 6 h and 17 h post-LPS stimulation conditions returned a list of 20 seed genes with the highest interactions. Among them, *Axl* was the most downstream effector (Figure 9A). Next, node neighborhood analyses and gene coexpression analysis suggested that BTEE not only affected the expression of AXL but also significantly downregulated several coexpressed and downstream gene expressions (Figure 9B–D). 

AXL is a member of the RTK family, the TAM receptors (TYRO3, AXL, MERTK) [31]. The *Axl* transcription process is also feedback controlled by other RTKs. Activated EGFR pathways and downstream MEK/ERK signaling were reported to promote AXL mRNA expression through the JUN transcription factor in a number of cancer cells [32]. In line with previous studies on AXL inhibition, we also found that a number of tyrosine kinases and their signaling pathways were significantly downregulated in BTEE-treated cells (Figure 7 and Figure 9E). Additionally, AXL activation is associated with phosphatidylinositol 3-OH kinase (PI3K) and its downstream targets Akt, MAP kinase, Stat, and NF-κB signaling pathways [33]. Our transcriptomic data showed that several of these *Axl* downstream pathways were significantly downregulated by BTEE treatment.

Several TFs that directly act on the AXL promotor were found to be enriched by the downregulated DEGs (Figure 9F), such as *Sp1*/*Sp3* and hypoxia-inducible factor 1α (*Hif1α*) [34]. 

Additionally, chemical/drug perturbation analysis of the downregulated DEGs showed several known *Axl* inhibitors, such as cabozantinib [35], iguratimod [36], fostamatinib [37], and lapatinib [38] (Figure 10A–D). Finally, gene–disease association enrichment analysis predicts the potential of BTEE in several inflammatory and skin diseases (Figure 10E). Altogether, our enrichment and network analyses suggest that the pattern of biological processes and pathways modulated by BTEE treatment is similar to those found in *Axl* inhibitors.

Growth arrest-specific 6 (GAS6), an AXL ligand, has been well reported to bind to the receptor and to initiate a series of AXL downstream signaling factors [39,40]. In our study, we could not find any effect of BTEE on *Gas6* expression. However, AXL activation does not depend on GAS6 ligand-dependent dimerization only; instead, it exhibited several activation patterns, including GAS6 ligand-independent dimerization, heterophilic dimerization of AXL with a TAM family member (like MER or TYRO3) or with a non-TAM family protein, and ligand-independent activation through transcellular homophilic binding [28]. Therefore, further studies need to clarify the mechanism of BTEE in interfering *Axl* activation.

Accumulating evidence supporting the role of AXL in inflammation, and cancer progression, metastasis, and treatment resistance [41,42,43,44,45], resulted in increased research attention on AXL inhibitors. Our study suggests that BTEE may exhibit anti-inflammatory effects by attenuating LPS-induced *Axl* expression.

One aspect that needs to be addressed is that we used murine macrophage RAW264 cell line, an established in vitro model used interchangeably as putative analogs to human macrophages [20,46], in our study to examine the anti-inflammatory activities of BTEE. However, since the targeted outcome for most anti-inflammatory pharmacological and biomaterial research and development is proven to have efficacy and safety in humans, further testing of BTEE in human cell lines and in vivo is necessary. 

Based on the above, BTEE is anticipated to be employed as a raw ingredient in nutraceuticals and functional foods to prevent or treat chronic inflammation as well as in cosmetics. According to our previous studies, the key components of BTEE include hydrocarbons, botryococcene, and methylated-meijicoccene, which have potential hair growth-promoting and anti-stress benefits [17,18]. We also examined the anti-inflammatory effects of botryococcene and methylated-meijicoccene on the LPS-mediated inflammatory responses in RAW264 cells; however, no significant effects were observed in the range of treatment concentrations of these compounds. Therefore, identifying the active compounds for the anti-inflammatory effects of BTEE is yet to be achieved. 

## 4. Materials and Methods

### 4.1. Preparation of BTEE

Lyophilized powder of BT TEPMO-26 (100 mg) was extracted with 1 mL of 70% Ethanol at room temperature and in the dark for two weeks [17]. After the extraction, the sample was centrifuged at 1000× *g* for 10 min, and the supernatant was collected. The extracted sample’s supernatant was filtered through a 0.22 µm filter unit and stored at −20 °C in the dark until use.

### 4.2. Cell Culture of RAW264 Cells

RAW264 cells (Resource No. RCB0535) were purchased from RIKEN BioResource Center (RIKEN BRC, Tsukuba, Ibaraki, Japan). RAW264 cells were cultured in Dulbecco’s modified Eagle medium (DMEM) (Merck KGaA, Darmstadt, Germany) supplemented with 10% heat-inactivated fetal bovine serum (FBS) (Bio West, Nuaillé, France) and penicillin-streptomycin solution (Merck KGaA, Darmstadt, Germany) at 37 °C in a humidified incubator containing 5% CO_2_ using 75 cm^2^ flask (BD FALCON, Corning, NY, USA).

### 4.3. MTT Assay

To evaluate the effect of BTEE on cell viability, the MTT assay was performed. MTT reagent, 3-(4,5-dimethylthiazol-2-yl)-2,5-diphenyl tetrazolium bromide, was purchased from Dojindo Inc. (Kumamoto, Japan) [9,10]. 

In brief, RAW264 cells were seeded into a 96-well plate at the density of 2.0 × 10^5^ cells per well and incubated at 37 °C overnight. The RAW264 cells were treated with or without BTEE at 37 °C for 24 h. After treatment, MTT reagent was added to each well (10 μL/well) and incubated at 37 °C for 24 h. Then, the formazan crystal was dissolved with 100 μL of 10% Sodium Dodecyl Sulfate (SDS) (Fujifilm Wako Pure Chemical Co., Osaka, Japan) in each well and incubated overnight at 37 °C. 

The absorbance was measured at 570 nm using a microplate reader, VARIOSKAN LUX (Thermofisher Scientific, Waltham, MA, USA). The values were normalized to the value of the medium and calculated as the percentage of control.

### 4.4. Preparation of LPS

LPS (*E. coli* O111:B4) was purchased from EMD Millipore Co. (Billerica, MA, USA). A total of 5 mg of LPS was dissolved in 2 mL of PBS (-) and stored at −80 °C in the dark until use. After the treatment, LPS solution (1 ng/mL) was added to each well and incubated for 16 h at 37 °C.

### 4.5. Measurement of NO Production

The Griess method measures the concentration of NO_2_ by measuring the absorbance of the diazo compound generated by the coupling reaction (Griess reaction) [9,10]. RAW264 cells were seeded into a 96-well plate at 2.0 × 10^5^ cells/mL density and incubated at 37 °C for 24 h. The cells were treated with or without BTEE at 37 °C for 24 h. 

After the BTEE treatment, the LPS solution (1 ng/mL) was added to each well and incubated for 16 h at 37 °C. Then, the supernatant of the cell culture medium was mixed 1:1 with the Griess reagent (1% sulfanilic acid and 0.1% N-(1-Naphthyl) ethylenediamine Dihydrochloride in 2.5% phosphoric acid). The absorbance was measured at 540 nm using a microplate reader (VARIOSKAN LUX, Thermofisher Scientific), and the nitrite concentration was determined by a dilution of sodium nitrite (NaNO_2_) as a standard. Sulfanilic acid, Phosphoric acid, and NaNO_2_ were purchased from Fujifilm Wako Pure Chemical Co. N-(1-Naphthyl) ethylenediamine Dihydrochloride was purchased from Tokyo Chemical Industry Co., Ltd., (Tokyo, Japan).

### 4.6. RT-qPCR Analysis of Gene Expression in RAW264 Cells

RT-qPCR was performed to evaluate the effect of BTEE on gene expression in RAW264 cells [10,15]. For the quantification of gene expression, the TaqMan probe was used. Total RNA was extracted using ISOGEN (Nippon Gene Co., Ltd., Tokyo, Japan) following the manufacturer’s instructions. RAW264 cells were seeded at 2.0 × 10^5^ cells/mL in 6-well dishes (BD FALCON, Corning) and incubated for 24 h at 37 °C. After that, cells were treated with or without 0.2 mg/mL BTEE and incubated for 24 h at 37 °C. After the treatment, LPS solution (1 ng/mL) was added to each well and incubated for 0, 6, or 17 h at 37 °C. After that, cells were lysed with 1 mL of ISOGEN. The lysates were obtained, and RNA was quantified using a Nanodrop 2000 spectrophotometer (Thermofisher Scientific). Next, reverse transcription reactions were carried out using SuperScript IV VILO Master Mix (Thermofisher Scientific) following the manufacturer’s instructions. For the quantification of amounts of transcripts, the TaqMan real-time RT-PCR amplification reactions were performed using the Applied Biosystems 7500 Fast Real-Time System (Thermofisher Scientific) following the manufacturer’s instructions. 

All the primer sets and TaqMan Universal PCR Master mix were purchased from Thermofisher Scientific. Specific primers Glyceraldehyde-3-phosphate dehydrogenase (*Gapdh*) (Mm99999915_g1), *Ccl2* (Mm00441242_m1), *Tnf* (Mm00443258_m1), *Cox2* (Mm00478374_m1), *iNos2* (Mm00440502_m1), *Il6* (Mm00446190_m1), *Il1b* (Mm00434228_m1), *Il10* (Mm01288386_m1), *Atf3* (Mm00476032_m1), *Socs1* (Mm00782550_s1), and *Pgc1β* (Mm00504730_m1) were used. Results of the mRNA levels of all genes were normalized using *Gapdh* as an internal control.

### 4.7. DNA Microarray Analysis for Gene Expression Profiling in RAW264 Cells

DNA microarray analysis was conducted following the manufacturer’s instructions provided by Thermofisher Scientific. In brief, as described above, total RNA from RAW264 cell was extracted using ISOGEN. Synthesis and in vitro transcription of complementary DNA (cDNA), purification and reverse transcription of cRNA, and synthesis, purification, fragmentation, and labelling of single-stranded cDNA (ss-cDNA) were performed using the GeneChip WT PLUS Reagent Kit (Thermofisher Scientific) following the manufacturer’s instructions. Cartridge Array Hybridization was performed using the cartridge array (Clariom S array, mouse; Thermofisher Scientific) on the GeneChip™ Fluidics Station (Thermofisher Scientific). Scanning was performed using the GeneChip Scanner (Thermofisher Scientific). Reagents from GeneChip™ Hybridization, Wash and Stain Kit were used for hybridization and scanning.

### 4.8. DNA Microarray Data Processing and Subsequent Analyses

The raw image data were normalized following the signal space transformation robust multi-chip analysis (SST-RMA) algorithm using the Transcriptome Analysis Console (TAC) software (ver. 4.0.2, Thermofisher Scientific). The SST-RMA method reduces background through GC Correction Version 4. Gene-level analysis was performed using the Limma Bioconductor package implemented in TAC software. One-way ANOVA followed by an empirical Bayes correction was performed for differential expression analysis. Detected Above Background (DABG) threshold was set to 0.05 and Pos/Neg area under the curve (AUC) threshold was set to 0.7. Finally, gene-level filter criteria were set as *p* value (one-way between-subject) <0.05 and fold change (in linear space) >1.5. Genes that passed the filter criteria were considered differentially expressed genes (DEGs). Subsequent analyses were performed using the DEGs between BTEE + LPS vs. LPS at 6 h (often refers to as 6H in the figures) and BTEE + LPS vs. LPS at 17-h conditions (often refers to as 17H in the figures). 

Gene ontology (GO) and pathway enrichment analyses were performed using the web-based tool Metascape v3.5.20230101 (http://metascape.org) (accessed on 8 January 2023) [47]. TRRUST [27] and DisGeNET [30] databases were used for TF and disease–gene association enrichment analyses, respectively. PPI networks were built using the InnateDB Interactome database [25] on the NetworkAnalyst tool version 3.0 (https://www.networkanalyst.ca/NetworkAnalyst/home.xhtml) (accessed on 22 December 2022) [48]. Heatmaps were generated using the Morpheus online tool (https://software.broadinstitute.org/morpheus/) (accessed on 22 December 2022). Kinases coexpression and chemical perturbation analyses were performed on the Enrichr tool (https://maayanlab.cloud/Enrichr/) (accessed on 22 December 2022) [49] using the ARCHS4 [26] and LINCS L1000 [29] data sets, respectively. 

### 4.9. Statistical Analysis

Results were expressed as mean ± standard deviations (SD). Student’s T-Test was used to determine the statistically significant difference between the means of the two groups. Graphs were prepared using Microsoft Excel software (version 2019).

## 5. Conclusions

In conclusion, we have evaluated the anti-inflammatory effects of the BTEE on the LPS-induced pro-inflammatory conditions in the RAW264 murine macrophage-like cells. Further investigation in human cell lines and identification of the active substance of BTEE will lead to the development of new anti-inflammatory agents derived from microalgae.

## Data Availability

The supporting data of this article can be found within this paper. The microarray data have been deposited in the NCBI GEO database and are publicly available (Accession number: GSE221665; https://www.ncbi.nlm.nih.gov/geo/query/acc.cgi?acc=GSE221665) (accessed on 23 December 2022).

## Supplementary Materials

The following supporting information can be downloaded at: https://www.mdpi.com/article/10.3390/ijms24076666/s1.
