# Supplementary material for: Botryococcus terribilis Ethanol Extract Exerts Anti-inflammatory Effects on Murine RAW264 Cells
Source: Int J Mol Sci. 2023 Apr 3;24(7):6666. doi: 10.3390/ijms24076666 (PMC10095501; doi:10.3390/ijms24076666)
Supplement: Supplementary file 1 [file ijms-24-06666-s001.zip › Captions.pdf]

**Figure S1:** Downregulated DEGs in BTEE-treated conditions related to 'cytokine–cytokine receptor interaction' KEGG pathway;

**Supplementary File S1:** Commonly regulated gene list;

**Supplementary File S2:** PPI network analysis of common downregulated DEGs;

**Supplementary File S3:** Kinase coexpression analysis by the downregulated DEGs;

**Supplementary File S4:** Drug/chemical perturbation analysis by the downregulated DEGs.
