# Supplementary material for: Botryococcus terribilis Ethanol Extract Exerts Anti-inflammatory Effects on Murine RAW264 Cells
Source: Int J Mol Sci. 2023 Apr 3;24(7):6666. doi: 10.3390/ijms24076666 (PMC10095501; doi:10.3390/ijms24076666)
Supplement: Supplementary file 1 [file ijms-24-06666-s001.zip › Supplementary Figure S1_Cytokine-cytokine receptor interaction-related gene expression.pptx]

## Slide 1
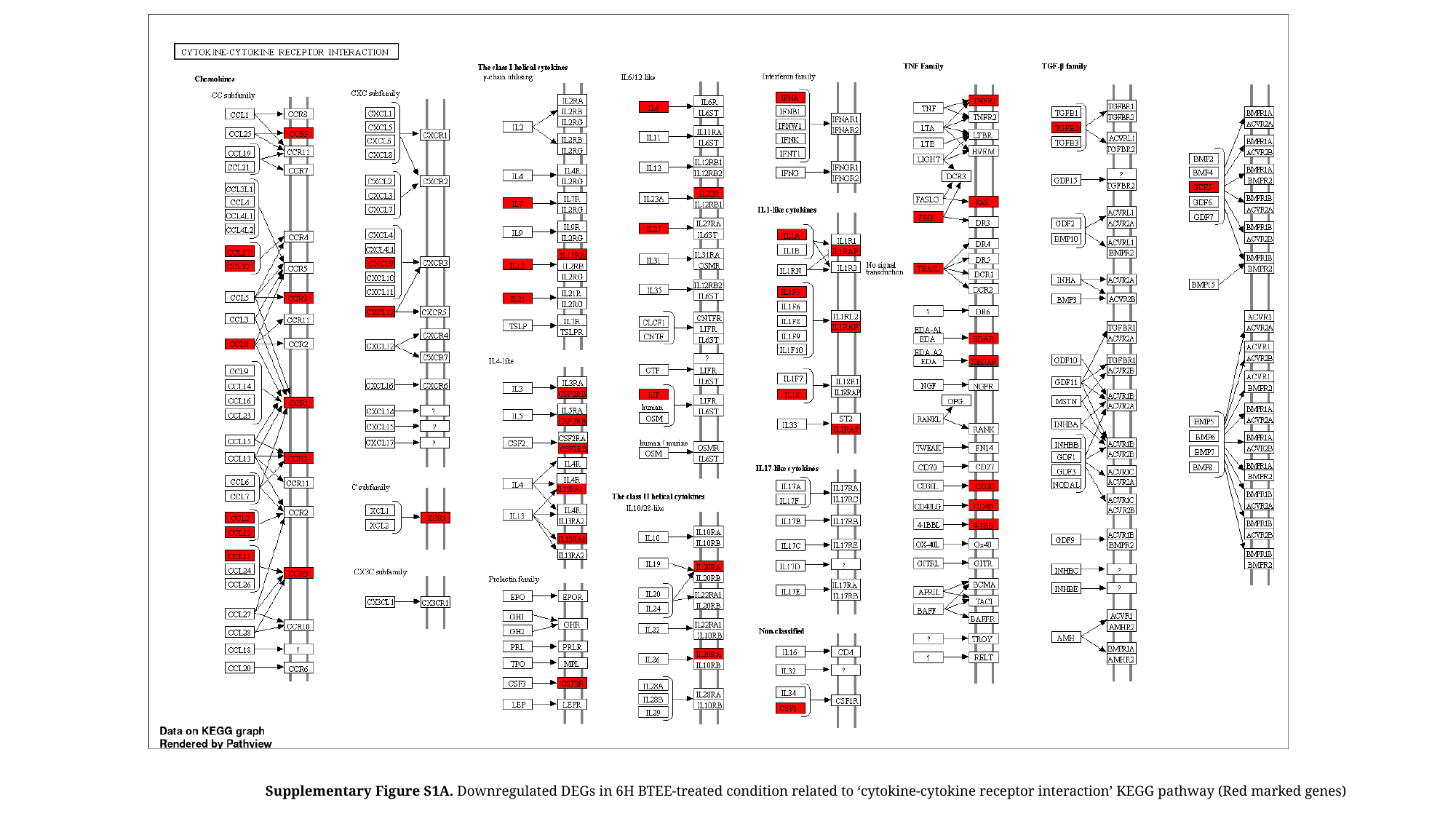

Supplementary Figure S1A. Downregulated DEGs in 6H BTEE-treated condition related to ‘cytokine-cytokine receptor interaction’ KEGG pathway (Red marked genes)

## Slide 2
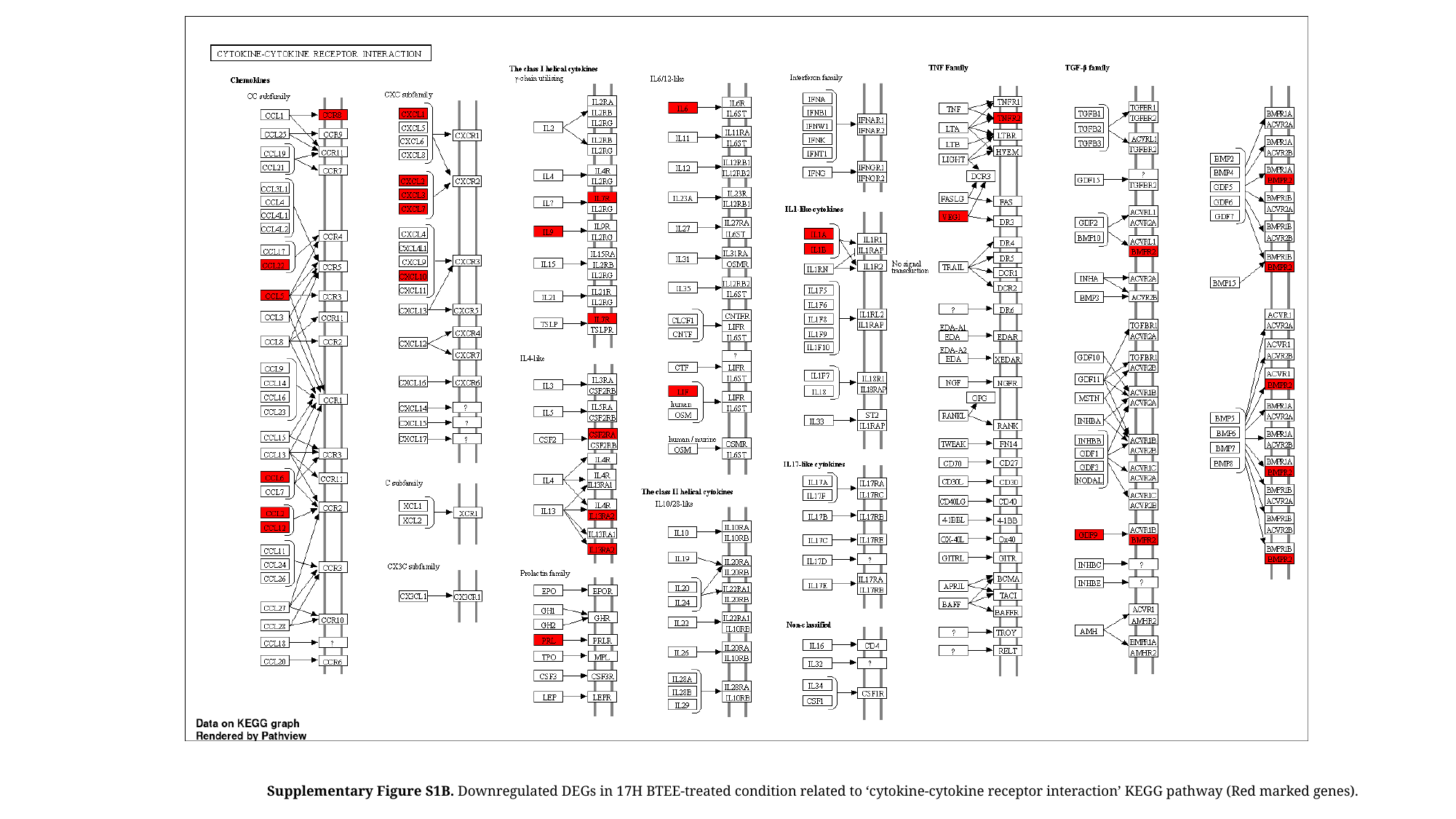

Supplementary Figure S1B. Downregulated DEGs in 17H BTEE-treated condition related to ‘cytokine-cytokine receptor interaction’ KEGG pathway (Red marked genes).
